# Rhizophagus clarus and phosphorus co-regulate oat rhizosphere metabolome–microbiome under drought: a candidate hub metabolite perspective

**DOI:** 10.3389/fpls.2026.1984943

**Published:** 2026-10-21

**Authors:** Bin Zhang, Jieyu Bao, Ze Zhang, Zixin Sun, Fusuo Zhang, Meijun Zhang

**Affiliations:** 1 College of Agriculture, Shanxi Agricultural University, Jinzhong, China; 2 College of Resources and Environmental Sciences, China Agricultural University, Beijing, China

**Keywords:** AMF, bacterial community, drought, metabolome, oat, phosphorus

## Abstract

The interaction between arbuscular mycorrhizal fungi (AMF) and phosphorus (P) availability could enhance plant drought tolerance. Yet whether this interaction modulates nutrient uptake and grain yield in oat by reshaping rhizosphere metabolite and microbial networks remains unresolved. A pot experiment was conducted across three relative water contents (75%, 55%, and 35% relative water content, designated WW, MDS, and SDS), two AMF treatments, and three P levels (0, 40, and 80 mg kg^−^¹ soil, designated P0, P1, and P2). Untargeted metabolomics and metagenomics were applied to characterize rhizosphere metabolite profiles and bacterial community structure, and to assess their relationships with nitrogen (N) and P status in the plant–soil system, grain yield and β-glucan. Drought significantly reduced grain yield, whereas moderate drought increased β-glucan accumulation; the beneficial effects of AMF on both traits were strongly modulated by soil P level. Under drought, AMF inoculation and P addition synergistically promoted N and P accumulation across oat organs; relative to P0AMF, P2AMF significantly elevated total N and P concentrations in roots, stems, leaves, panicles, and grains. Water availability was the dominant driver of rhizosphere metabolome and bacterial community variation. Under drought, AMF–P interaction further enriched rhizosphere metabolites, including amino acids and derivatives, organic acids, and benzene and substituted derivatives, while shifting the signature bacterial taxa toward *Pseudomonadota* (P1AMF) and *Myxococcota* and *Gemmatimonadota* (P2AMF). Network analysis identified isopentenyl pyrophosphate (IPP) as a highly connected hub metabolite, exhibiting the strongest associations with N and P nutrition, grain yield, β-glucan, and bacterial community composition. Exogenous IPP application significantly increased oat plant height, stem dry weight, and average root diameter under drought and markedly elevated the relative abundances of several rhizosphere bacterial taxa. Collectively, these findings indicate that AMF inoculation combined with P addition is associated with restructuring of the rhizosphere metabolite–microbe network under drought, and that IPP represents a candidate metabolite that warrants further investigation in these plant–microbe–soil interactions. This study provides a correlative framework linking this dual strategy to shifts in rhizosphere microbial communities and crop yield and functional quality under water-limited conditions, although mechanistic validation will require future experimental work.

## 1 Introduction

The rhizosphere is a dynamic soil–plant interface where root-secreted metabolites simultaneously function as chemotactic signals for beneficial microorganisms and selective filters enriching microbial taxa with functional traits (Zhalnina et al., 2018; Chen et al., 2022; Roldán et al., 2025). These recruited microorganisms can further compensate for functional gaps in the host genome, such as biological N fixation and P mobilization, thereby improving soil N and P use efficiency (Jiang et al., 2021; Wang et al., 2023; Cernava, 2024; Qiao et al., 2024). In turn, bioactive molecules released by the microbial community are associated with systemic plant responses, which dynamically refine microbiome structure and functional specificity through feedback regulation (Qiu et al., 2025). The resulting ensemble of primary and secondary metabolites secreted by plants and their associated symbionts into the rhizosphere collectively constitutes the chemical currency driving rhizosphere interactions (Douglas, 2020), forming a cross-kingdom communication network that facilitates nutrient acquisition and stress adaptation (Swenson et al., 2018; Wen et al., 2022).

Although total soil P can be substantial, only a small fraction is readily available to plants, as P is strongly retained through adsorption, precipitation, and incorporation into organic forms (Holford, 1997; Richardson and Simpson, 2011). Its inherently low bioavailability and poor mobility severely constrain plant acquisition, particularly under drought stress (Meisser et al., 2019), making the enhancement of soil P use efficiency a central goal for sustainable dryland agriculture. Plants access soil P through two principal routes: the direct pathway via root epidermal cells and root hairs, and the mycorrhizal pathway via AMF hyphae (Smith and Read, 2008; Brundrett and Tedersoo, 2018). AMF form symbiotic associations with over 80% of terrestrial plant species, and the mycorrhizal pathway alone can account for more than half of total plant P uptake (Smith and Read, 2008). In exchange, host plants typically allocate up to 20% of their photosynthate, primarily carbohydrates and fatty acids, to sustain hyphal network development and symbiotic stability (Keymer et al., 2017; Genre et al., 2020), with the mycorrhizal contribution to crop P uptake being substantial under favorable conditions (Chu et al., 2020). Beyond direct P transfer, AMF extraradical hyphae serve as dispersal conduits for bacteria toward organic P-enriched soil zones, enhancing bacterial access to and utilization of organic P (Jiang et al., 2021). The hyphosphere has accordingly been described as the “second genome” of AMF, capable of amplifying plant P uptake by modulating P-cycling functions of the associated microbiome (Zhang et al., 2022). Crucially, improved P nutrition itself is a key determinant of plant drought tolerance (Zeng et al., 2025), underscoring the broad potential of the AMF–plant symbiosis for simultaneously enhancing P use efficiency and mitigating drought stress. Despite the recognized roles of *Rhizophagus clarus* in phosphorus uptake and drought mitigation (Oliveira et al., 2022; Silva et al., 2023), how P modulates its symbiotic benefits under water deficit remains unresolved, motivating the present factorial study. Furthermore, the contribution of AMF to nutrient transformation has been shown to depend substantially on ambient soil nutrient levels (Chu et al., 2020), and key rhizosphere metabolites, including flavonoids, soluble sugars, fatty acids, and polyamines, have been implicated in AMF-mediated stress tolerance networks (Sheteiwy et al., 2021; Zou et al., 2021, Zou et al., 2023), positioning metabolomics and microbiome profiling as powerful tools for dissecting mycorrhizal mechanisms under stress.

Oat (*Avena sativa* L.) is an annual Poaceae crop encompassing naked oat (*Avena sativa* concv. *nuda*) and hulled oat (*Avena sativa* subsp*. sativa*) (Ahmad et al., 2014). The β-glucan content in its grains directly influences the added value of deeply processed products, rendering oat a crop of considerable interest in the food processing and pharmaceutical industries (Sikora et al., 2013). In China, naked oat dominates, constituting approximately 90% of the national cultivation area and serving primarily as a food staple. China ranks among the world’s leading oat-producing nations (FAOSTAT, http://www.fao.org/faostat/en/, accessed December 2025). Oat-producing regions in China are concentrated in the arid to semi-arid agro-pastoral ecotone between 40°N and 46°N, where mean annual precipitation is approximately 400 mm (Liu et al., 2011), rendering oat highly susceptible to drought throughout its growth cycle (Chen et al., 2026). Beneficial plant–microbe interactions, particularly mutualistic symbioses, can alleviate drought by enhancing nutrient use efficiency (Tian et al., 2023), and AMF are known to form functional symbioses with oat (Cornejo et al., 2010; Lee et al., 2023; Cao et al., 2024). Despite this, how AMF–P interactions under drought shape rhizosphere metabolite and microbial composition in oat, and how these rhizosphere components synergistically mediate oat growth, β-glucan accumulation, and N and P use efficiency, remains poorly understood.

To address these gaps, pot experiments simulating drought via controlled irrigation were conducted with oat under combined AMF inoculation and P addition treatments. The specific objectives were to: (1) determine the interactive effects of AMF inoculation and P addition on soil N and P availability and their distribution across oat organs under drought; (2) characterize how AMF and P differentially shape rhizosphere metabolite profiles and bacterial community structure under drought, and clarify their relationships with N and P nutrition, grain yield and β-glucan; and (3) identify candidate metabolites within the AMF- and P-mediated rhizosphere symbiotic network and evaluate their potential for regulating oat growth and rhizosphere microbial communities. This work provides a correlative framework linking AMF–P interactions to rhizosphere metabolome–microbiome shifts, generating testable hypotheses for future mechanistic studies aimed at improving oat yield and quality in rainfed systems.

## 2 Materials and methods

### 2.1 Experimental site and design

The experiment was conducted in the dry shed of Shanxi Agricultural University, Jinzhong, China (37°42′13″N, 112°57′10″E; altitude 797 m) from March to June 2024.

A completely randomized three-factor design was employed, yielding 18 treatment combinations. The first factor comprised three soil water levels: well-watered (WW; 75% relative water content), moderate drought stress (MDS; 55%), and severe drought stress (SDS; 35%). The second factor was P addition rate (as P_2_O_5_): P0 = 0, P1 = 40, and P2 = 80 mg kg^−^¹ soil. The third factor was AMF inoculation: 60 g active inoculant (AMF) or 60 g sterilized inoculant (NAMF control). Each treatment had 18 replicate pots, with sampling at the jointing, heading, and maturity stages. At each stage, six pots per treatment were destructively sampled: three for AMF root colonization rate and grain yield, and three for organ and rhizosphere soil collection. The experiment totaled 324 pots. An additional unplanted pot under MDS with AMF inoculation was established as a non-rhizosphere soil control.

Each pot received 3.5 kg experimental soil amended with the designated P level and a basal N dose of 0.315 g urea (CH_4_N_2_O; 46% N). Inoculant or sterilized inoculant was thoroughly mixed with the bottom two-thirds of the soil prior to seeding. Seedlings were thinned to six plants per pot at the three-leaf stage. Pots were weighed daily at 18:00 (CST) and watered to maintain the target soil water content.

### 2.2 Experimental materials

The locally prevalent oat cultivar Baiyan 2 was used throughout. Prior to sowing, uniform full-grain seeds were surface-sterilized with 80% carbendazim, then soaked and germinated.

Experimental soil was collected from the 0–25 cm tillage layer and classified as Typic Haplustalfs (USDA Soil Taxonomy), with the following baseline properties: pH 7.10, organic matter 19.13 g kg^−^¹, ammonium-N 9.24 mg L^−^¹, nitrate-N 10.17 mg L^−^¹, available P 8.67 mg kg^−^¹, and field water capacity 34.02%. Air-dried soil was passed through a 4 mm sieve and blended with river sand (10:1, w/w). To remove indigenous AMF, a portion of soil was autoclaved at 121 °C for 2 hours. To restore the non-AMF microbial community in sterilized soil, an AMF-free microbial filtrate was prepared by suspending 500 g unsterilized soil in 5000 mL sterile water and filtering through six layers of quantitative filter paper (pore size 16 μm; Wang et al., 2013), which retains AMF spores and hyphae while passing general soil microbiota. This filtrate was applied to sterilized soil at 10 mL per pot.

The AMF species used was *R. clarus*, obtained from the Beijing Academy of Agriculture and Forestry Research. Inoculant was produced by cultivating *R. clarus* on clover (*Trifolium* spp.) roots in autoclaved sand under controlled conditions for four months, after which shoot material was removed and the remaining pot contents, comprising spores, extraradical hyphae, colonized root segments, and sandy soil, were used as inoculant. Clover mycorrhizal colonization reached 92.82%, and each 10 g of inoculant contained 74 spores. A parallel batch of inoculant was autoclaved to serve as the NAMF control.

### 2.3 Sample collection and analyses

Grain yield was determined at maturity. Water use efficiency for grain yield (WUE*_Y_*) was calculated as the ratio of grain yield to total water consumed over the entire growth period (Batool et al., 2019):


$${\text{WUE}}_{\text{Y}}\left({\text{g L}}^{-1}\right)=\text{Grain Yield}/\text{Quantity of Irrigation Water}$$

For plant tissue sampling, six plants per pot were harvested at each growth stage and separated into stems, leaves, spikes (heading), spike axes (heading and maturity), glumes (maturity), and grains (maturity). The β-glucan content in oat grains at maturity was determined using a specific β-glucan assay kit (K-BGLU, Megazyme, Ireland) manufactured by Megazyme. The aboveground organ separation scheme is illustrated in **Supplementary Figure S1**. Tissues were oven-killed at 105 °C for 30 minutes, dried to constant weight at 80 °C, and ground to a fine powder using a TissueLyser II cell disruptor (QIAGEN, Hilden, Germany) for total N and P analysis. For rhizosphere soil sampling, aboveground biomass was removed, bulk and surface soil were discarded, and soil adhering within 2 mm of the root surface was collected and homogenized as the rhizosphere sample. Fresh subsamples were stored at room temperature, −20 °C, or −80 °C for soil nutrient analysis, untargeted metabolomics, and metagenomics, respectively. Analytical methods for N and P concentrations in plant organs and rhizosphere soil, and soil pH, are detailed in the **Supplementary Material**.

### 2.4 Untargeted metabolomics

Metabolite profiling was performed on rhizosphere soil samples collected at the heading stage, selected on the basis of the soil chemical property results described above. Briefly, 250 mg of soil was transferred to a centrifuge tube with 500 μL of 70% methanol (pre-cooled to −20 °C), vortexed for 3 minutes, and further homogenized with steel beads for an additional 3 minutes if necessary. Samples were then sonicated in an ice-water bath for 10 minutes, vortexed for 1 minute, and held at −20 °C for 30 minutes. After centrifugation at 12, 000 rpm for 10 minutes, the supernatant was filtered through a 0.22 μm PTFE membrane and stored at 4 °C prior to analysis. All samples were analyzed by two complementary LC-MS methods; detailed HPLC and MS parameters are provided in the **Supplementary Material**. All raw files are archived in OMIX (accession OMIX019388, BioProject PRJCA071079), the miscellaneous omics data repository of the National Genomics Data Center, National Center for Bioinformation (CNCB-NGDC), Chinese Academy of Sciences.

### 2.5 Metagenomic sequencing

Total DNA was extracted from the rhizosphere soil samples using the DNeasy PowerSoil Pro Kit (QIAGEN, Germany) according to the manufacturer’s protocol. The concentration and integrity of the extracted DNA were assessed using the Qubit^®^ dsDNA Assay Kit on a Qubit^®^ 4.0 Fluorometer (Life Technologies, CA, USA) and a Qsep400 Bioanalyzer, respectively. Only DNA samples with a concentration sufficient to provide ≥ 0.2 μg total input and a main peak size greater than 500 bp were used for subsequent library construction. For each qualified sample, 0.2 μg of input DNA was sheared to approximately 300 bp fragments. The sheared fragments were then end-repaired, A-tailed, and ligated with Illumina sequencing adapters (Illumina, San Diego, CA, USA), followed by PCR amplification. The amplified products were purified, and the size distribution of the final libraries was verified on a Qsep400 Bioanalyzer. The pooled libraries were denatured and loaded onto a sequencing flow cell. Sequencing was performed on an Illumina NovaSeq platform using a PE150 strategy, with an average depth of approximately 8.0 Gb of clean data per sample. All raw sequencing data generated in this study have been deposited in the NCBI Sequence Read Archive (SRA) under the accession number PRJNA1470768.

### 2.6 Oat phenotype response to isopentenyl pyrophosphate

A separate pot experiment was conducted under MDS using sterilized soil prepared as described above. A completely randomized two-factor design was applied, crossing IPP treatment (IPP *vs*. NIPP) with planting status (planted, _P; unplanted, _NP), yielding four treatment combinations (IPP_P, IPP_NP, NIPP_P, NIPP_NP) each with three replicates (12 pots total). IPP standard was purchased from Alta Scientific Co., Ltd. (Tianjin, China). Each IPP-treated pot received 2 mL of 0.5 mM IPP solution, while NIPP controls received an equal volume of sterile water. The IPP concentration (0.5 mM) was selected based on a preliminary dose-range screening (0.1, 0.5, 1, and 2.0 mM), in which 0.5 mM promoted seedling growth without visible phytotoxicity, whereas higher concentrations caused mild stress symptoms under the same experimental conditions. Planted pots were thinned to six plants per pot at the three-leaf stage. After 30 days, shoot and root biomass were determined and root morphology was quantified using WinRHIZO software (Regent Instruments Inc., Québec City, Canada). Rhizosphere soil was collected for 16S rRNA gene amplicon sequencing; details are provided in **Supplementary Material**.

### 2.7 Statistical and bioinformatic analysis

AMF colonization rate, grain yield, β-glucan content, organ nutrient concentrations, and rhizosphere soil nutrient content were analyzed by three-way ANOVA with Duncan’s multiple range test in DPS 9.01 data processing system. Figures were produced in OriginPro 2021 (OriginLab, Northampton, MA, USA).

For metabolomics quality control, pooled QC samples were prepared from all sample extracts and injected every 10 samples; instrument stability was confirmed by Pearson correlation of QC replicates (|r| → 1) using the base package (v4.1.2) in R. Principal component analysis (PCA) was applied to explore overall variation in rhizosphere metabolome and microbial community composition using the same base package. Orthogonal partial least squares discriminant analysis (OPLS-DA), implemented in the MetaboAnalyst package (v1.0.1) in R, was used to identify differential metabolites while minimizing confounding variance, with differential selection criteria set at VIP > 1.0 and fold change > 2.0. Unigenes were annotated against bacterial sequences extracted from the NCBI NR database using DAMND (v0.9.9; Brundrett and Tedersoo, 2018; blastp, e-value ≤ 1×10^−5^). Hierarchical clustering of metabolites across all 90 samples was conducted with the ComplexHeatmap package (v2.12.0) in R.

Stacked bar plots of bacterial phylum- and species-level relative abundances were generated with ggplot2 (v3.3.5) in R. Per-sample gene abundance correlation heatmaps were produced using the psych package (v2.2.9) in R. Discriminative taxa were identified by LEfSe analysis (LDA score threshold 3.7; Segata et al., 2011). To characterize associations among rhizosphere metabolites, bacterial communities, and plant and soil indicators, a network heatmap was constructed via https://cloud.metware.cn, integrating Spearman correlations among differential metabolites and bacterial taxa with Mantel test-based associations among indicators, metabolites, and microbial communities, implemented using the varSelRF package (v0.7.8) in R. Metabolic pathway enrichment for metabolites linked to microbial community composition and key indicators was performed against the KEGG database (https://www.kegg.jp/) and visualized as bubble plots.

## 3 Results

### 3.1 AMF colonization rate, grain yield, β-glucan content, and WUE_Y_

*R. clarus* successfully colonized oat roots across all water and P treatments (**Figures 1a, e**), though drought progressively suppressed colonization. Peak colonization occurred under WW at P0, reaching 50.33%, 95.67%, and 79.33% at the jointing, heading, and maturity stages, respectively (**Figures 1a, e**). A significant AMF × P interaction was detected for both grain yield and β-glucan content, with both traits declining under severe drought stress (**Figures 1b, c**). P1 significantly boosted yield relative to both P0 and P2 under WW and MDS, while AMF inoculation produced its greatest yield benefit at P0, increasing yield by 12.61%, 35.80%, and 400.00% over NAMF across the three water regimes, respectively (**Figure 1b**). Compared with P0, both P1 and P2 treatments significantly increased grain β-glucan content under both WW and MDS conditions. AMF inoculation further promoted grain β-glucan accumulation, with increases of 6.42% (P1) and 6.81% (P2) under WW, and 8.97% (P1) and 10.93% (P2) under MDS, relative to the NAMF treatment (**Figure 1c**). Water use efficiency for grain yield (WUE*_Y_*) followed a rise-then-fall pattern across water regimes, peaking at 72.38% in the MDS P1AMF treatment (**Figure 1d**).

**Figure 1:**
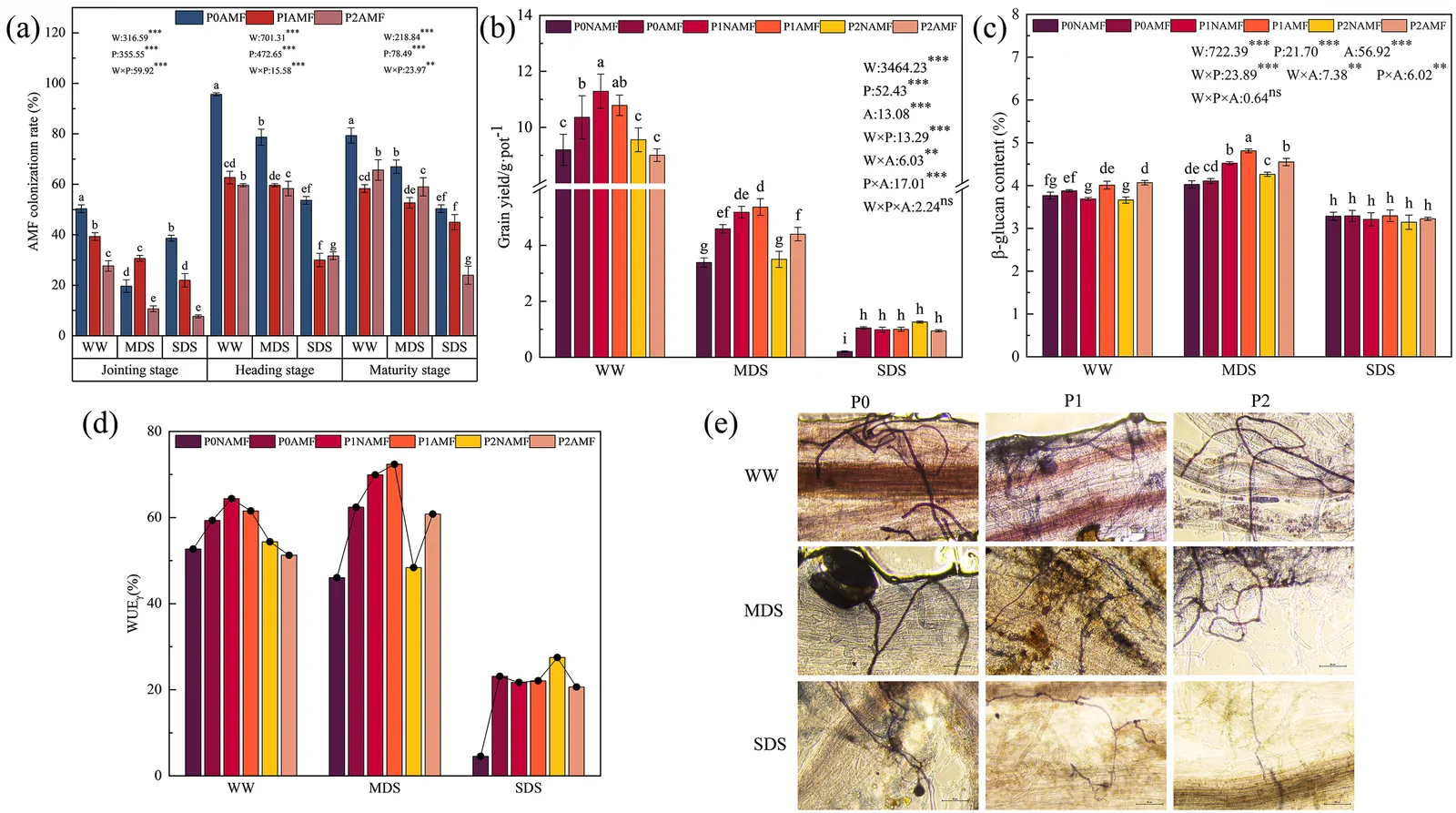
AMF colonization **(a)**, grain yield **(b)**, β-glucan content **(c)**, water use efficiency (WUE*_Y_*) **(d)**, and AMF-infected roots (scale bar = 50 μm) **(e)** of oat under drought stress caused by arbuscular mycorrhizal fungi (AMF) inoculation in combination with phosphorus (P) addition. The values indicate the means ± standard deviations (n = 3). Different letters in the same column indicate significant differences among treatments (*P<* 0.05). W, A, and P represented water treatment, AMF, and phosphorus treatment, and the following values are *F* values. ns indicates no significant difference, * indicates significant at the 0.05 level, ** indicates significant at the 0.01 level and *** indicates significant at the 0.001 level. WW, well-watered; MDS, moderate drought stress; SDS, serious drought stress; P0, 0 mg kg^−1^ soil; P1, 40 mg kg^−1^ soil; P2, 80 mg kg^−1^ soil; NAMF, AMF-free; AMF, AMF inoculation.

### 3.2 Rhizosphere soil N, P, and pH

Drought markedly altered rhizosphere soil nutrient status across all three growth stages. Relative to WW, SDS elevated rhizosphere concentrations maximally by 41.93% for total N, 198.72% for ammonium-N, 181.87% for nitrate-N, 24.94% for total P, and 106.65% for available P (**Supplementary Tables S1**, **S2**). MDS, by contrast, reduced total P by 15.27% relative to WW, and soil pH declined significantly with decreasing water availability (**Supplementary Table S2**).

P addition did not significantly affect total N, ammonium-N, or nitrate-N, but strongly influenced total P, available P, and pH. P2 produced maximum increases of 26.56% in total P and 171.54% in available P relative to P0, and reduced soil pH by 11.26%; P1 had no detectable effect on pH (**Supplementary Table S2**). Compared with NAMF, AMF inoculation reduced nitrate-N and soil pH by up to 13.55% and 4.46%, respectively, while increasing available P by up to 29.47% (**Supplementary Tables S1**, **S2**).

The combined AMF × P interaction did not significantly affect total N or ammonium-N but did reduce soil pH. Notably, under drought (MDS and SDS), nitrate-N and available P increased progressively with P application rate. P2AMF yielded the greatest nitrate-N enrichment, with maximum increments of 28.37% under MDS and 24.61% under SDS relative to P0AMF (**Supplementary Table S3**). Available P responded even more strongly, with P2AMF producing peak increases of 186.99% under MDS and 216.76% under SDS (**Supplementary Table S4**).

### 3.3 Total N and P in oat organs

Drought substantially reduced N and P accumulation across all oat organs over the three growth stages. Under SDS, total N content declined relative to WW by up to 16.67% in roots, 14.51% in stems, 50.39% in leaves, 9.33% in spikes, 24.70% in spike axes, 30.87% in glumes, and 39.82% in grains; corresponding total P reductions reached 51.56%, 64.56%, 73.95%, 41.27%, 38.94%, 45.88%, and 21.58%, respectively (**Supplementary Tables S5**, **S6**).

P addition significantly influenced root total N and spike total P at the heading stage, with P2 increasing these by 10.40% and 24.44% relative to P0, respectively (**Supplementary Tables S5**, **S6**). AMF inoculation redistributed nutrients among organs: relative to NAMF, AMF reduced total N maximally by 13.23% in roots, 17.10% in stems, and 9.57% in spike axes (**Supplementary Table S5**), while increasing total P by up to 32.26% in roots, 43.48% in stems, 54.69% in leaves, 17.89% in spikes, and 6.77% in grains (**Supplementary Table S6**).

The combined AMF × P effects on organ N and P were drought-dependent. Under MDS, P2AMF significantly raised total N in all organs and total P in leaves and spikes relative to P0AMF, with peak increases of 15.94% and 23.36%, respectively (**Supplementary Tables S7**, **S8**). Under SDS, P2AMF significantly elevated total N in roots, leaves, glumes, and grains, and total P in all measured organs, with maximum increases of 10.59% and 62.86%, respectively (**Supplementary Tables S7**, **S8**).

### 3.4 Rhizosphere soil metabolite composition

QC replicates clustered tightly in PCA across all samples, confirming instrument stability and data reproducibility (**Supplementary Figure S2**). Twenty-one metabolite classes were detected in the rhizosphere soil, dominated by amino acids and derivatives (20.6%), benzene and substituted derivatives (11.17%), organic acids (9.92%), alcohols and amines (7.49%), and glycerophospholipids (GP; 5.57%) (**Supplementary Figure S3**). PC1 clearly separated samples by water regime (WW vs. MDS vs. SDS; **Supplementary Figure S4**), identifying water availability as the primary driver of rhizosphere metabolome variation. OPLS-DA confirmed distinct metabolite profiles across water treatment groups (**Supplementary Figure S7a**), P treatment groups (**Supplementary Figure S7c**), AMF inoculation groups (**Supplementary Figure S7e**), AMF × P combinations under drought (**Figure 2a**), and AMF × water regime groups, including the unplanted NR control (**Supplementary Figure S5**). All validation models showed high predictive accuracy (R²Y > 0.95) and robustness (Q² > 0.85; **Supplementary Figures S7b, d, f**, **Figure 2b**), confirming that treatment-induced metabolomic differences were both statistically significant and analytically reliable.

**Figure 2:**
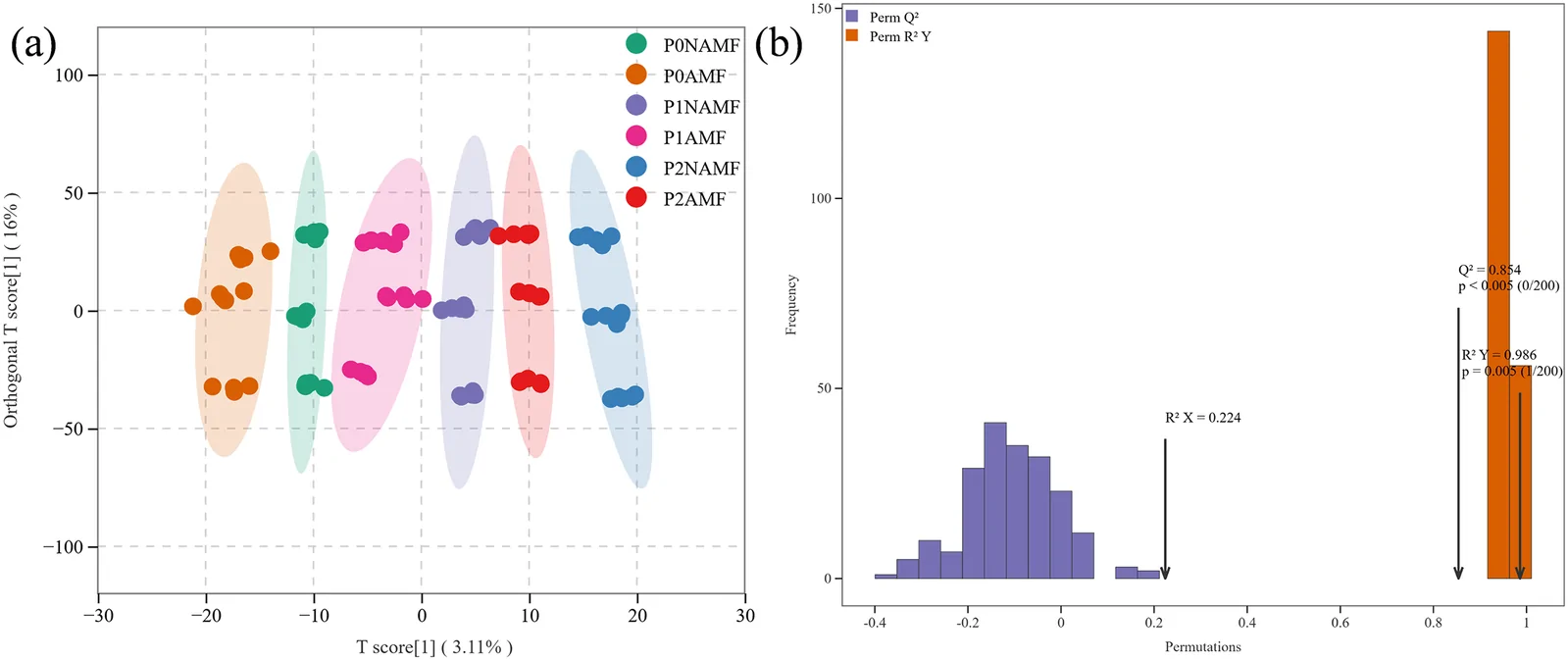
OPLS-DA score plot **(a)** and validation model **(b)** of metabolites in oat rhizosphere soil caused by arbuscular mycorrhizal fungi (AMF) inoculation in combination with phosphorus (P) addition under drought stress. Component 1 and 2 account for inter-group and intra-group variations, respectively, with their corresponding eigenvalues indicating the percentage of total variance explained in the dataset. The ellipse reflects the 95% confidence interval of samples in each group. P0, 0 mg kg^-1^ soil; P1, 40 mg kg^-1^ soil; P2, 80 mg kg^-1^ soil; NAMF, AMF-free; AMF, AMF inoculation.

Cluster heatmap analysis revealed that 13 key metabolites, spanning amino acids and derivatives, organic acids, alkaloids, benzene and substituted derivatives, and flavonoids, were significantly more abundant under WW than under MDS or SDS (**Supplementary Figure S8a**, **Supplementary Table S9**). Under P0, rhizosphere metabolite abundance was markedly higher than under P1 or P2, particularly for benzene and substituted derivatives, amino acids and derivatives, nucleotides and derivatives, and lipids (**Supplementary Figure S8b**, **Supplementary Table S10**). AMF-inoculated rhizosphere soil showed significantly greater abundance of organic acids, amino acids and derivatives, alcohols and amines, lipids, flavonoids, fatty acids, and heterocyclic compounds relative to NAMF (**Supplementary Figure S8c**, **Supplementary Table S11**). Under drought, P1AMF and P2AMF rhizospheres were enriched in benzene and substituted derivatives, and GP compared to P0AMF, whereas P0AMF exhibited higher heterocyclic compounds, alkaloids, lipids, alcohols, and amines (**Supplementary Figure 3**, **Supplementary Table S12**). Venn diagram analysis of differential metabolites across water regimes and P levels identified 67 core metabolites shared among WW, MDS, and SDS comparisons, of which amino acids and derivatives (18), organic acids (14), and benzene and substituted derivatives (8) were the most represented classes (**Supplementary Figure S6**, **Supplementary Table S13**).

### 3.5 Bacterial community composition in oat rhizosphere soil

Microbial α-diversity varied significantly among treatments. Both Chao1 and Shannon indices were higher in AMF-inoculated rhizospheres than in NAMF controls, and under WW relative to MDS and SDS (**Supplementary Figure S9**), while P addition (P1 and P2) had comparatively little effect on these metrics. Inter-sample unigene abundance correlation coefficients approached unity, confirming high between-replicate similarity and experimental reliability (**Supplementary Figure S10**). PCA clearly separated bacterial community compositions by water regime (WW vs. drought) and by AMF inoculation status (**Supplementary Figures S11a, c**).

At the phylum level, *Pseudomonadota* and *Actinomycetota* jointly dominated the rhizosphere bacterial community, collectively accounting for over 65% of sequences across all samples (**Supplementary Figures S12a–c**; **Figure 3a**). Under drought, the relative abundance of *Actinomycetota* was consistently higher in all AMF-inoculated treatments (P0AMF, P1AMF, P2AMF) than in their respective NAMF counterparts (**Figure 3a**). At the species level, water regime, P level, AMF inoculation, and the AMF × P combination each shaped the composition of the top-ten bacterial taxa in the rhizosphere (**Supplementary Figures S12d–f**; **Figure 3b**). Relative to the unplanted NR control, AMF inoculation combined with varying water regimes substantially restructured bacterial community composition at both phylum and species levels (**Supplementary Figure S13**).

**Figure 3:**
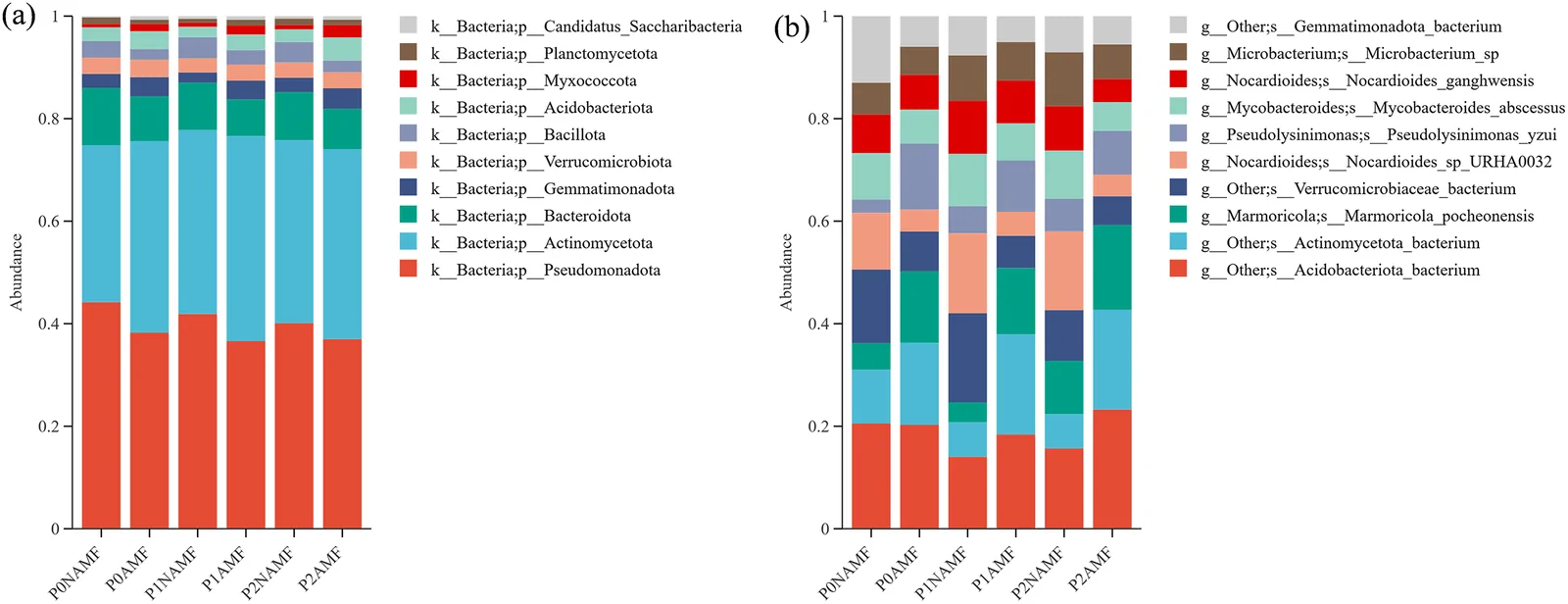
The difference of the top 10 bacterial community composition at the phylum level **(a)** and species level **(b)** caused by arbuscular mycorrhizal fungi (AMF) inoculation in combination with phosphorus (P) addition under drought stress. P0, 0 mg kg^−1^ soil; P1, 40 mg kg^−1^ soil; P2, 80 mg kg^−1^ soil; AMF, AMF inoculation.

LEfSe analysis further resolved treatment-specific marker taxa (**Supplementary Figure S14**). *Actinomycetota* and *Pseudomonadota* were the primary indicator phyla under both MDS and SDS, whereas *Gemmatimonadota* and *Verrucomicrobiota* were exclusive markers of WW (**Supplementary Figure S14a**). *Bacteroidota* was uniquely indicative of P0, and *g_Archangium* was the sole marker species under P2 (**Supplementary Figure S14b**). *Pseudomonadota* was the dominant marker phylum in both NAMF and AMF treatments, yet the two groups diverged at finer taxonomic levels: *Bacteroidota* and *Bacillota* were exclusive to NAMF, while *Myxococcota* and *Acidobacteriota* characterized AMF (**Supplementary Figure S14c**). Within the AMF × P combinations, *Actinomycetota* was uniquely associated with P0AMF, *Pseudomonadota* exclusively defined P1AMF, and only family *f_Sphingomonadaceae* marked P2NAMF. *Bacillota* and *Bacteroidota* were distinctive of P1NAMF, whereas P2AMF was uniquely characterized by *Myxococcota* and *Gemmatimonadota* (**Figure 4**).

**Figure 4:**
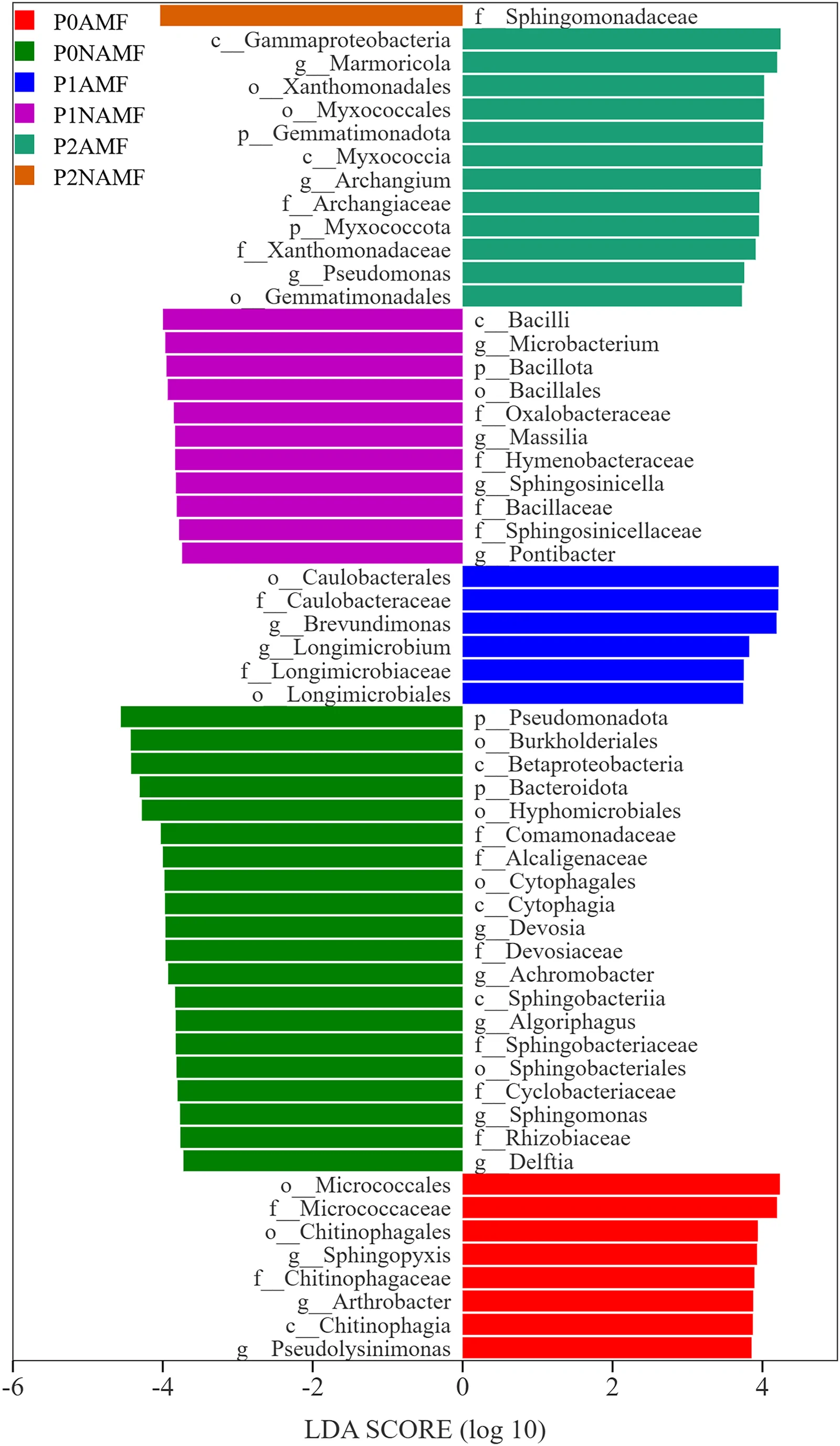
LEfSe analysis of bacterial community composition in response to arbuscular mycorrhizal fungi (AMF) inoculation in combination with phosphorus (P) addition under drought stress. P0, 0 mg kg^−1^ soil; P1, 40 mg kg^−1^ soil; P2, 80 mg kg^−1^ soil; NAMF, AMF-free; AMF, AMF inoculation.

### 3.6 Associations among plant–soil nutrient status, grain yield, β-glucan, rhizosphere metabolites, and bacterial community

Spearman correlation and hierarchical clustering of differentially abundant features were used to map metabolite–microbiome associations (**Supplementary Figure S15**). In the water regime comparison, Cluster I (**Supplementary Figure S15a**, **Supplementary Table S14**) comprised 26 metabolites and 8 bacterial taxa; metabolites were dominated (62%) by amino acids and derivatives, benzene and substituted derivatives, organic acids, and alkaloids, and the bacterial component included *Bacillota* and *Actinomycetota*. Cluster II (**Supplementary Figure S15a**, **Supplementary Table S14**) contained 24 metabolites and 14 bacterial taxa, with 83% of metabolites drawn from amino acids and derivatives, alkaloids, GP, and benzene and substituted derivatives, alongside *Pseudomonadota*, *Bacteroidota*, *Verrucomicrobiota*, *Gemmatimonadota*, and *Acidobacteriota*. In the AMF comparison, Cluster I (**Supplementary Figure S15b**, **Supplementary Table S14**) included 24 metabolites, 18 positively correlated, and *Actinomycetota*, with 56% of metabolites belonging to terpenoids, lignans and coumarins, benzene and substituted derivatives, and amino acids and derivatives. In the P-level comparison, Cluster I (**Supplementary Figure S15c**, **Supplementary Table S14**) contained 13 metabolites dominated (56%) by benzene and substituted derivatives, terpenoids, alkaloids, and amino acids and derivatives, whereas Cluster II (**Supplementary Figure S15c**, **Supplementary Table S14**) contained 13 metabolites with 70% from amino acids and derivatives, benzene and substituted derivatives, heterocyclic compounds, lipids, and phenolic acids. In the AMF × P drought comparison, Cluster I (**Supplementary Figure S15d**, **Supplementary Table S14**) encompassed 28 metabolites and 28 bacterial taxa; metabolites were 64% from amino acids and derivatives, heterocyclic compounds, lipids, and organic acids, with bacterial taxa spanning *Acidobacteriota*, *Actinomycetota*, *Gemmatimonadota*, *Verrucomicrobiota*, *Pseudomonadota*, *Bacteroidota*, *Chloroflexota*, *Planctomycetota*, and *Myxococcota*. Cluster II (**Supplementary Figure S15d**, **Supplementary Table S14**) comprised 16 metabolites and 22 bacterial taxa, with 56% of metabolites from amino acids and derivatives, benzene and substituted derivatives, organic acids, and GP, alongside *Actinomycetota*, *Pseudomonadota*, *Bacteroidota*, and *Verrucomicrobiota*.

Mantel test analysis revealed that N and P nutrition, grain yield, and β-glucan content were significantly associated with bacterial community composition, and that rhizosphere metabolites were strongly correlated with soil available P, pH, spike N, and spike P, positioning these variables at the nexus of both microbial and metabolomic variation (**Figure 5a**). Network reconstruction confirmed that the majority of bacterial taxa were highly connected to key indicators (**Figure 5b**). Within this network, IPP (MEDP1614; classified as terpenoid) emerged as the central hub metabolite, carrying the greatest number of connections to N and P nutrient status, grain yield, β-glucan, and bacterial community composition (**Figure 5b**; **Supplementary Tables S14**, **S15**).

**Figure 5:**
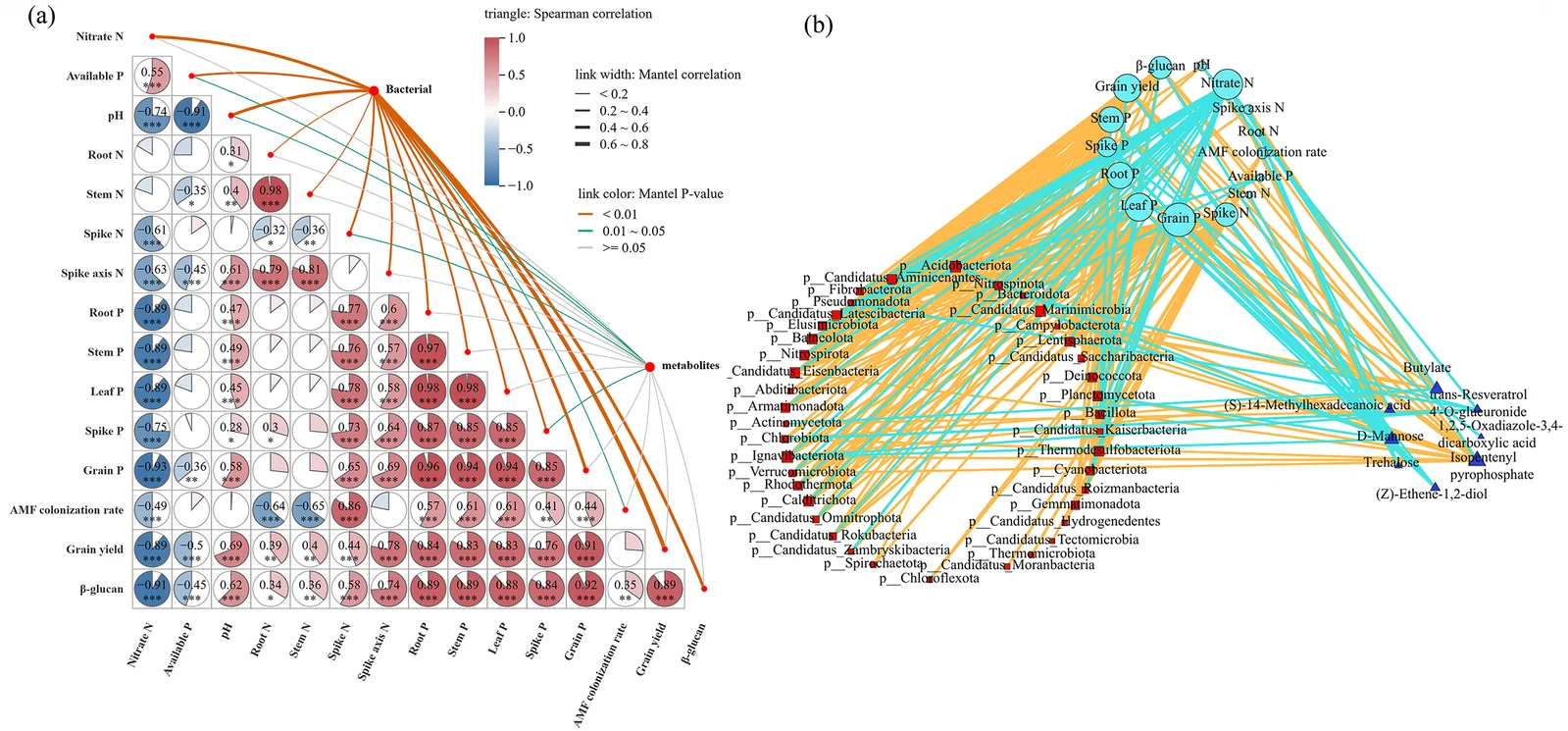
Correlations among the nutrients state in plant and soil, grain yield, metabolites, and bacterial community. **(a)** Relationships between key indicator, bacterial community, and metabolites. The lower triangular area shows the Spearman correlations between the key indicator. The size of the colored blocks within the cells represents the correlation coefficient, with red indicating positive correlations and green indicating negative correlations. * indicates significant at the 0.05 level, ** indicates significant at the 0.01 level and *** indicates significant at the 0.001 level. The upper triangular area displays Mantel test results between each indicator and bacterial community/metabolites. The line thickness represents the overall correlation coefficient, while the line color indicates the significance level of the correlation. **(b)** Symbiotic networks between the key indicator, the top 50 soil metabolites, and the top 50 species of bacteria at the phylum level. The size of the nodes in the network indicates the number of connections. A connection indicates a strong and significant correlation. Dark orange and turquoise lines represent positive and negative relationships (Spearman’s correlation, *P* < 0.05, r > 0.720), respectively.

### 3.7 Oat root morphology, biomass, and rhizosphere bacterial community response to exogenous IPP

Relative to the no-IPP control, exogenous IPP significantly increased average root diameter, plant height, stem dry weight, and leaf dry weight by 22.73%, 10.77%, 7.45%, and 6.11%, respectively, while root length, root surface area, and root dry weight declined by 36.64%, 25.61%, and 6.71%, respectively (**Table 1**). Under planted conditions, IPP addition significantly elevated the rhizosphere soil Shannon index and increased the relative abundances of *Proteobacteria*, *Gemmatimonadota*, and *Bacteroidota* compared to the IPP-free control (**Figures 6a–c**). Under unplanted conditions, IPP treatment did not significantly alter the Shannon index, but did significantly increase the relative abundance of the core dominant phylum Firmicutes. Significant between-group differences were also observed for four low-abundance rare phyla, *Gracilibacteria*, *Kapabacteria*, *Nanoarchaeota*, and *Elusimicrobiota* (**Figures 6a, b, d**).

**Table 1:** Isopentenyl pyrophosphate (IPP) effects on oat root morphology and phenotype.

Treatment	Length (cm)	Surface area (cm^2^)	Average diameter (mm)	Volume (cm^3^)	Plant height (cm)	Root dry weight (g)	Stem dry weight (g)	Leaf dry weight (g)
NIPP_P	1408.53a	195.09a	0.44b	2.16a	51.73b	1.49a	0.94b	1.31b
IPP_P	892.47b	145.12b	0.54a	1.88a	57.30a	1.39b	1.01a	1.39a
*P*	< 0.05	< 0.05	< 0.05	ns	< 0.001	< 0.01	< 0.01	<0.01
F	12.93	12.38	15.40	3.98	753.76	36.60	26.94	50.01

The values indicate the means (n = 3). Different letters in the same column indicate significant differences among treatments (*P* < 0.05). ns indicates no significant difference. NIPP_P and IPP_P indicate the absence and addition of IPP under planted conditions, respectively.

**Figure 6:**
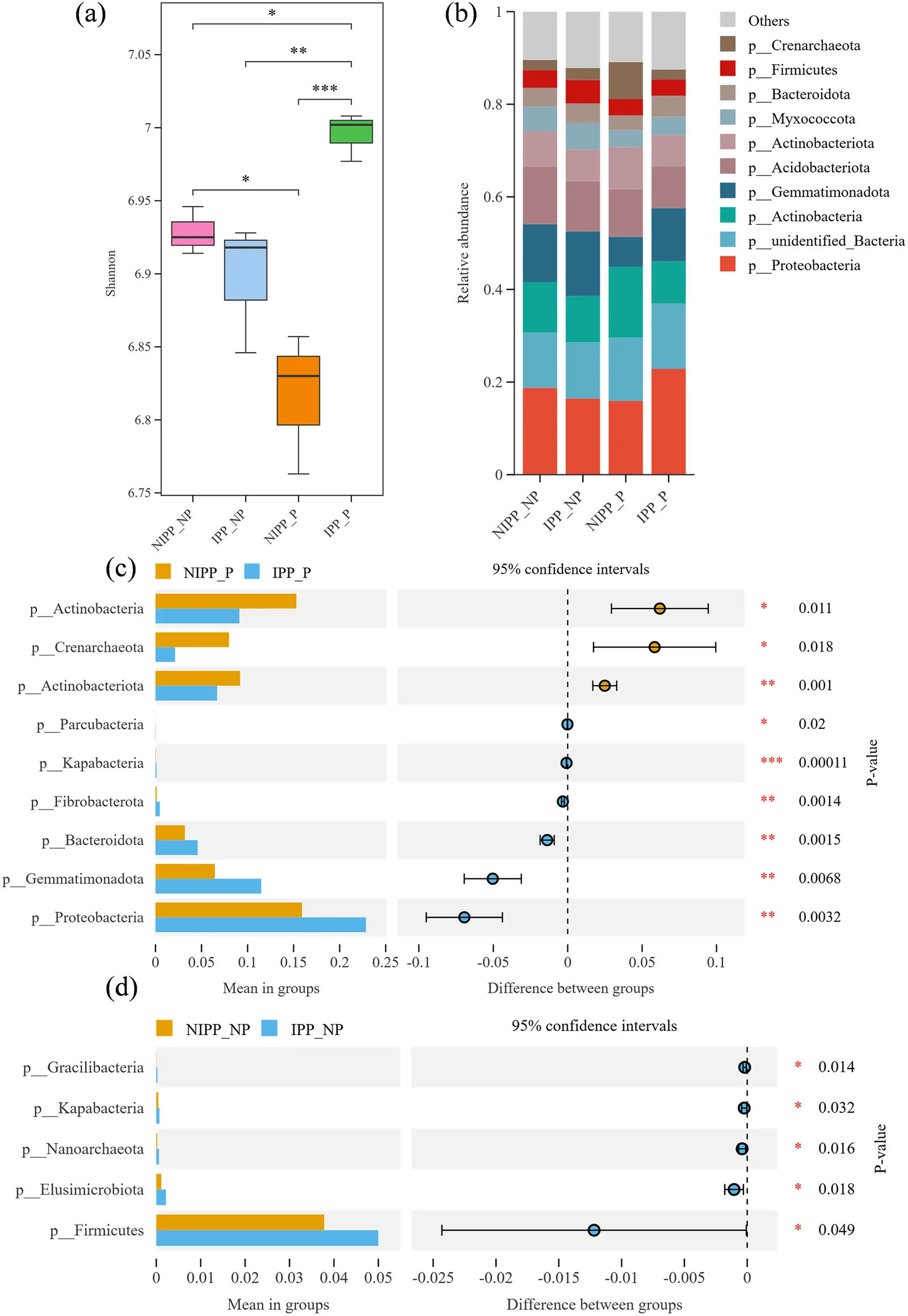
Isopentenyl pyrophosphate (IPP) effects on bacterial community in oat rhizosphere soil. **(a)** Shannon index of soil bacterial communities under different treatments. **(b)** Relative abundance of dominant bacterial phyla in each treatment; **(c)** Significantly different bacterial phylum between NIPP_P and IPP_P treatments; **(d)** Significantly different bacterial phylum between NIPP_NP and IPP_NP treatments. * indicates significant at the 0.05 level, ** indicates significant at the 0.01 level and *** indicates significant at the 0.001 level. NIPP_P and IPP_P indicate the absence and addition of IPP under planted conditions, respectively. NIPP_NP and IPP_NP indicate the absence and addition of IPP under unplanted conditions, respectively.

## 4 Discussion

### 4.1 AMF and moderate P addition mediate nutrient redistribution as a key pathway for increasing oat grain yield and β-glucan under drought

Effective AMF colonization and extraradical hyphal development are central to water and nutrient acquisition in host plants (Tang et al., 2022). Here, both drought stress and elevated P supply significantly suppressed AMF colonization of oat roots (**Figures 1a, e**). Drought-induced reductions in photosynthetic carbon fixation may constrain carbon allocation to the root–AMF symbiosis, thereby contributing to the observed reduction in AMF colonization (Wang et al., 2021). Concurrently, high soil available P allows plants to satisfy P demand through direct uptake, diminishing their dependence on AMF and reducing C allocation to the symbiont (Ven et al., 2019; Wen et al., 2019). Together, these responses point to a dynamic “trade balance” between oat and AMF, wherein C-for-P exchange is continuously recalibrated according to prevailing drought intensity and P availability.

Drought consistently reduced grain yield irrespective of AMF inoculation, while moderate P addition (P1) effectively boosted yield under both WW and MDS (**Figure 1b**). These results accord with Zhang et al. (2025), who documented persistent drought-induced yield suppression in AMF-inoculated oat, and with Das et al. (2022), who found that moderate-to-high P application substantially offset drought-driven yield losses in AMF-inoculated rice. Notably, grain yield reached its maximum at P1 rather than P2, despite greater P availability at the higher rate (**Supplementary Table S1**; **Figure 1b**), suggesting a threshold beyond which additional P no longer benefits yield. This pattern may be attributed to a nonlinear yield response of oat to P supply, as evidenced by the greater yield response to P1 than to P2, with additional P beyond P1 conferring limited benefit in the present study. Under SDS, however, P addition failed to improve yield, whereas AMF inoculation without P (P0AMF) did produce a significant yield increase (**Figure 1b**). This apparent paradox likely reflects severe drought-induced physiological disruption: stomatal closure and metabolic suppression under extreme water deficit (Tanveer et al., 2023; Yang et al., 2025) transiently impair P uptake and degrade the functional benefit of AMF symbiosis. The significantly higher WUE*_Y_* under WW and MDS relative to SDS (**Figure 1d**) lends further weight to this interpretation. Additionally, the present study found that under both WW and MDS conditions, P addition significantly increased grain β-glucan content, and inoculation with AMF further amplified this promoting effect (**Figure 1c**). This result indicates a synergistic interaction between P and AMF symbiosis in regulating β-glucan metabolism. As a core element in energy metabolism and signal transduction, P addition can enhance the activities of sucrose synthase and UDP-glucose pyrophosphorylase (Li et al., 2022), thereby providing an adequate substrate supply for β-glucan synthesis (Fogarty et al., 2020). In addition, AMF may indirectly influence β-glucan accumulation by improving plant P acquisition, carbon assimilation and overall source–sink balance. Collectively, these findings indicate that pairing an optimal P rate with AMF inoculation offers a practical strategy for moderating drought-induced losses in both grain yield and β-glucan content in oat, particularly where moderate rather than severe drought predominates.

Prolonged drought significantly lowered rhizosphere soil pH and diminished soil N and P availability, ultimately constraining oat nutrient uptake capacity (**Supplementary Tables S1**-**S6**). Drought-driven increases in root organic acid exudation transiently acidify the rhizosphere and help mobilize insoluble nutrients (Xu et al., 2021; Wang et al., 2025), yet this adaptive response provides only short-lived improvements in nutrient bioavailability (Xiao et al., 2020). More consequentially, drought disrupts the “root exudation–microbial activity–soil chemistry” cascade (Karlowsky et al., 2018; Veach et al., 2020), setting in motion a self-reinforcing cycle of soil acidification → nutrient immobilization → uptake inhibition. The unexpected accumulation of nitrate N under drought stress further suggests that reduced plant uptake, together with a passive soil concentration effect, may jointly outweigh the drought-induced decline in nitrification. Additionally, altered microbial nitrogen fixation and attenuated denitrification in drought-affected soils may also contribute to this accumulation process. Targeted manipulation of the rhizosphere microenvironment therefore represents a critical leverage point for breaking this negative feedback loop (Roldán et al., 2025).

P addition significantly modified rhizosphere soil total P, available P, and pH, and regulated N partitioning to roots and P allocation to panicles at the heading stage (**Supplementary Tables S1**-**S6**). As an essential structural component of ATP, P provides the energetic substrate for high-demand physiological processes such as grain filling (Hu et al., 2024), and directs preferential nutrient allocation to reproductive organs through its interconnected roles in energy supply (Cabeza et al., 2024), signal transduction (Lu et al., 2026), and hormonal regulation (Hasan et al., 2016). Beyond P supply alone, AMF-mediated nutrient redistribution significantly enhanced nitrate-N and available P mobility throughout the plant–soil system, steering these nutrients toward reproductive organs (**Supplementary Tables S1**-**S6**), with a notably stronger effect on P than N. This asymmetry reflects the evolutionary specialization of AMF as “P specialists, “ equipped with dedicated P acquisition mechanisms including organic acid secretion and expansive hyphal networks (Ferrol et al., 2019), whereas plants retain predominant control over N assimilation via direct root uptake of NO_3_^−^ and NH_4_^+^ (Bücking and Kafle, 2015). AMF achieves this redistribution through a three-pronged mechanism: physical expansion of the absorptive network (Qin et al., 2023), biochemical mobilization of fixed nutrients (Monokrousos et al., 2020; Qiu et al., 2022), and signal-guided reallocation of plant resources (Boyno et al., 2023). These findings collectively establish AMF inoculation combined with P addition as an effective agronomic lever for optimizing N and P allocation to reproductive organs during critical growth stages, with direct implications for yield formation under drought.

### 4.2 AMF and low P addition as key drivers of rhizosphere metabolome variation under drought

Rhizosphere metabolites, encompassing plant root exudates, microbial metabolic products, and background soil C compounds, play central ecological roles in shaping microbial community composition and directing succession dynamics (Baker et al., 2024; Fan et al., 2025). The oat rhizosphere metabolome proved highly complex, spanning 21 chemical classes, with amino acids and derivatives predominating, followed by benzene and substituted derivatives and organic acids (**Supplementary Figure S3**). Amino acids are among the nitrogenous compounds that can be exchanged between AMF and host plants (Li et al., 2025). In the rhizosphere, amino acids can originate from multiple sources, including root exudation, microbial metabolism, and decomposition of soil organic matter (Schimel and Bennett, 2004; Ma et al., 2022). Specific amino acids such as tryptophan also function as chemotactic signals that recruit beneficial rhizosphere bacteria, including *Pseudomonas* (Meng et al., 2025). Benzene and substituted derivatives encompass a chemically diverse group of compounds, some of which may participate in plant defense or carbon-related processes (Hättenschwiler and Vitousek, 2000; Thevenot et al., 2010), while organic acids are integral to nutrient mobilization and energy metabolism (Mustonen and Oelbermann, 2025).

Drought stress significantly suppressed oat growth in concert with a systemic decline in the abundance of multiple key rhizosphere metabolites (**Supplementary Figures S4**, **S7a**, **S8a**, **Supplementary Table S10**). The drought-responsive metabolites fell into two broad functional groups: basic metabolism-related compounds (amino acids and derivatives, organic acids) and plant defense-related compounds (alkaloids, benzene and substituted derivatives, flavonoids). This coordinated metabolic shift reflects a well-recognized chemical ecological strategy: under resource limitation, plants reprioritize basic metabolic maintenance by redirecting C sources while simultaneously adjusting defense compound biosynthesis to maximize survival. This pattern is consistent with the classical model in which drought systematically restructures the soil–plant metabolic network through a cascade of photosynthetic inhibition → C limitation → metabolic pathway reprogramming (Bernardo et al., 2019; Martínez-Peña et al., 2023), and provides fresh metabolomic evidence for the molecular basis of crop–soil interactions under water stress.

P is indispensable for ATP and NADPH synthesis, and its deficiency forces plants to redirect metabolism toward secondary pathways to preserve C skeleton pools (Dissanayaka et al., 2021). Accordingly, P0 significantly elevated the abundance of key rhizosphere metabolites relative to P1 and P2, with particularly pronounced increases in benzene and substituted derivatives, amino acids and derivatives, nucleotides and derivatives, and lipids (**Supplementary Figures S7c**, **S8b**, **Supplementary Table S10**). This likely reflects a multi-pronged adaptive response to P starvation, comprising metabolic pathway switching, resource substitution, and upregulated stress defense, wherein survival is prioritized over growth (Plaxton and Tran, 2011; López-Arredondo et al., 2014; Veneklaas, 2022).

The AMF–plant symbiosis is co-regulated through C–nutrient exchange, microbial interactions, and metabolic pathway activation (Gutjahr and Parniske, 2013; Kaur and Suseela, 2020). Relative to NAMF, AMF inoculation significantly elevated the abundance of organic acids, amino acids and derivatives, alcohols and amines, lipids, flavonoids, fatty acids, and heterocyclic compounds in the rhizosphere (**Supplementary Figures S7e**, **S8c**, **Supplementary Table S11**). In the absence of AMF, plants tend to channel C resources toward structural defense compounds such as lignin rather than diversifying secondary metabolite production (Pozo et al., 2015), resulting in functional homogenization of the rhizosphere microbiome and a contracted rhizosphere metabolic network. Notably, the metabolite profile of AMF-inoculated oat under drought differed markedly from that of the unplanted NR control (**Supplementary Figure S5**), a divergence attributable to the absence of plant-derived C in the NR treatment, which relegates AMF to dormancy or minimal metabolic activity (Smith and Read, 2008) and leaves soil organic matter decomposition as the primary metabolite source, producing a simpler, less diverse metabolic profile overall. These findings suggest that AMF may function not only as nutrient-acquisition partners but also as important modulators of rhizosphere chemical composition.

Under drought, the effects of AMF and P addition on rhizosphere metabolite composition were both independent and synergistic (Wang et al., 2023). P1AMF and P2AMF significantly enriched benzene and substituted derivatives and GP relative to P0AMF, while P0AMF conversely showed higher abundances of heterocyclic compounds, alkaloids, lipids, alcohols, and amines (**Figures 2**, **7**; **Supplementary Table S11**). The enrichment of these metabolite classes may reflect altered carbon allocation and plant–microbe interactions associated with AMF colonization, although the underlying metabolic pathways remain to be resolved (Keymer et al., 2017). Under P deficiency, the enrichment of N-containing metabolites may alter microbial resource availability and thereby contribute to the recruitment or selection of particular microbial taxa (Chaudhary et al., 2025).

**Figure 7:**
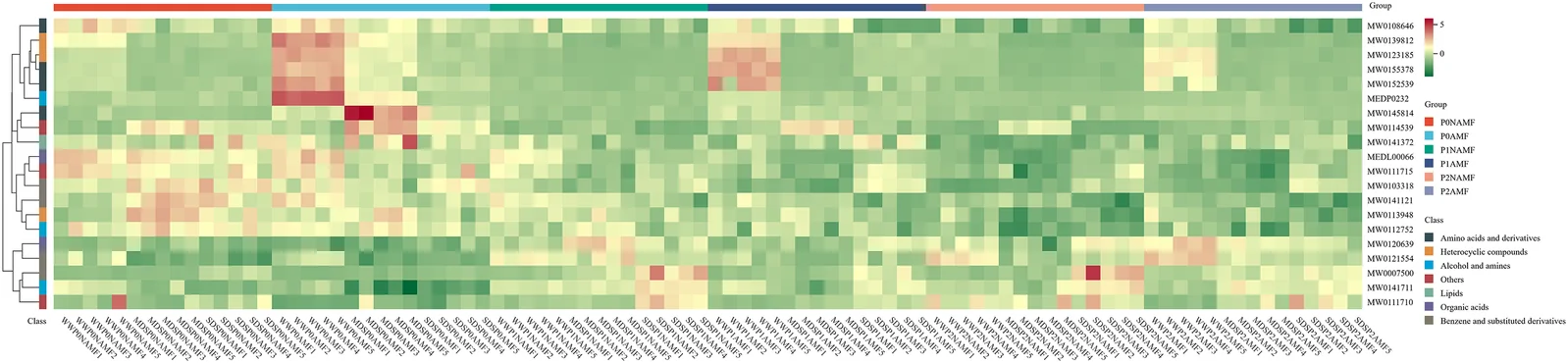
Cluster heatmap of the top 20 differential metabolites in oat rhizosphere soil caused by arbuscular mycorrhizal fungi (AMF) inoculation in combination with phosphorus (P) addition under drought stress. The heatmap displays a matrix of the relative abundance of the sample name (columns) and metabolite identifiers (rows). Group assignments are indicated by color bars, while the color gradient represents z-score normalized abundance values (red: high abundance; blue: low abundance). P0, 0 mg kg^−1^ soil; P1, 40 mg kg^−1^ soil; P2, 80 mg kg^−1^ soil; NAMF, AMF-free; AMF, AMF inoculation.

### 4.3 AMF and P addition promote bacterial enrichment in the oat rhizosphere under drought

The dominant bacterial phyla identified in oat rhizosphere soil, namely *Pseudomonadota* and *Actinomycetota*, mirror those reported in the rhizosphere microbiomes of various vegetable crops (Song et al., 2024; Wang et al., 2024). These two phyla may operate synergistically with AMF through complementary P-supply roles: AMF sustains microbial activity via C provision, *Pseudomonadota* mobilizes soluble P to release readily available forms (Alharbi et al., 2025), and *Actinomycetota* continuously liberates slow-release P through organic matter decomposition (Wang et al., 2023), together establishing a gradient P supply system. Relative to the unplanted NR control, combined AMF inoculation and water treatment substantially restructured dominant bacterial phyla and shifted taxon abundances at both phylum and species levels. This divergence likely reflects the NR community reverting toward a background “starvation” state in which copiotrophic bacteria predominate and community structure is governed primarily by soil physicochemical properties rather than plant-mediated biological interactions (Zhou and Ning, 2017).

Both water regime and AMF inoculation significantly reshaped rhizosphere bacterial community composition (**Supplementary Figure S11**). LEfSe analysis identified *Actinomycetota* and *Pseudomonadota* as the indicator phyla for MDS and SDS, while *Gemmatimonadota* and *Verrucomicrobiota* were uniquely enriched under WW (**Supplementary Figure S14a**). *Pseudomonadota* was a shared biomarker across both NAMF and AMF groups; however, *Bacteroidota* and *Bacillota* were exclusive to NAMF, whereas *Myxococcota* and *Acidobacteriota* specifically characterized AMF-inoculated rhizospheres (**Supplementary Figure S14c**). P addition, by contrast, exerted no significant effect on community structure differentiation (**Supplementary Figure S11c**), with *Bacteroidota* emerging as the sole biomarker phylum for P0 and *g_Archangium* showing selective enrichment under P2 (**Supplementary Figure S14b**). This contrast between water/AMF and P effects reflects a fundamental mechanistic difference: water availability and AMF shape bacterial communities through direct stress, resource allocation, and niche competition (Berruti et al., 2016; Das et al., 2022; Zhu et al., 2023), whereas phosphatase synthesis and H_2_PO_4_^−^ uptake are broadly distributed across *Proteobacteria*, *Actinomycetota*, and other phyla (Richardson and Simpson, 2011). The near-universal distribution of P-solubilizing capacity in the microbiome implies that P addition primarily modulates the expression of P-related functional genes rather than restructuring community membership.

A consistent pattern across treatments was the higher relative abundance of *Actinomycetota* in all AMF-inoculated treatments (P0AMF, P1AMF, P2AMF) compared to their NAMF counterparts (**Figure 4**). AMF hyphae release sugars and organic acids into the rhizosphere (Yadav et al., 2021), preferred C sources for *Actinomycetota*, and even under P addition, AMF continued to partially rely on the phosphate-solubilizing capacity of *Actinomycetota* (Wakelin et al., 2012). In NAMF treatments, plants met P demand largely through direct root uptake (Raphael et al., 2022), leaving *Actinomycetota*’s P-mobilizing potential underutilized. The enrichment of simple carbon substrates may have favored fast-growing copiotrophic taxa, potentially contributing to the lower relative abundance of *Actinomycetota* observed in NAMF treatments (Zhalnina et al., 2018).

It should be noted that the metagenomic analysis in this study primarily focused on taxonomic composition and biomarker identification, and did not include direct functional annotation, such as KEGG pathway mapping, CAZyme profiling, or quantitative detection of key P-cycling genes (e.g., *phoD*, *phoX*, *pstSCAB*, *ppk*) and N-cycling related genes. Therefore, the functional significance regarding enhanced nutrient transformation and phosphorus cycling was inferred indirectly from soil and plant nutrient concentrations as well as rhizosphere metabolite profiles, without direct gene-level evidence. Future studies should employ metagenomic approaches combined with functional annotation or targeted gene quantification to validate these functional interpretations.

### 4.4 Linking plant–soil nutrient status, grain yield, β-glucan, and bacterial community to hub metabolites

Taxon-specific responses to individual rhizosphere metabolites are a major driver of rhizosphere bacterial community assembly (Baker et al., 2024). Spearman correlation analysis and hierarchical clustering of differentially abundant features revealed tight associations between rhizosphere metabolites, principally amino acids and derivatives and benzene and substituted derivatives, and specific bacterial phyla including *Bacillota* and *Actinomycetota*, forming functionally distinct metabolite–microbiota interaction clusters across water, P, and AMF treatments (**Supplementary Figure S15**, **Supplementary Table S14**). These tightly coupled relationships reflect the three-way dynamic among plants, microbes, and environment: plants chemically steer rhizosphere microbiota through targeted metabolite secretion; microbial communities reciprocally modulate plant metabolite biosynthesis and exudation; and environmental factors, including water, P, and AMF, act as selective pressures that maintain the system’s dynamic equilibrium. This framework not only deepens mechanistic understanding of rhizosphere microecology but also identifies potential molecular targets for the directed engineering of rhizosphere microbial communities.

Mantel test analysis revealed a significant coupling relationship between bacterial community composition and grain yield, β-glucan, and nutrient variables. This finding strongly suggests that N and P nutrition not only serve as direct substrates for plant growth and metabolism but may also indirectly influence yield and grain quality by driving the assembly of distinct rhizosphere bacterial communities, which in turn modulate nutrient acquisition and metabolic signaling. Critically, our analysis identified soil available P, pH, spike N, and spike P were tightly coupled to both bacterial community composition and soil metabolite profiles (**Figure 5a**). This is consistent with the conceptual framework proposed by Bardgett and Van Der Putten (2014), in which soil properties exert selective pressure → microbial functional adaptation → metabolite response → plant nutritional feedback, and aligns with emerging perspectives on soil nutrient availability as a primary driver of rhizosphere microbial assembly (Zhang et al., 2024; Adeniji et al., 2025). This interconnected network underscores that effective agricultural ecosystem management must address soil chemistry, microbial function, and plant physiology as an integrated system rather than as isolated variables.

Within the association network, IPP emerged as the most highly connected metabolite, carrying the greatest number of connections to oat organ N and P status, yield components, and bacterial community composition (**Figure 5b**; **Supplementary Table S14**). Exogenous IPP application significantly promoted oat growth and enhanced rhizosphere microbial enrichment (**Table 1**; **Figure 6**), providing preliminary evidence that IPP can influence plant and microbial phenotypes under controlled conditions. IPP is a central precursor of plant isoprenoid metabolism and contributes to the biosynthesis of diverse terpenoids and hormone-related compounds (Vranová et al., 2013; Xi et al., 2016). In the present study, IPP showed the highest network connectivity and its exogenous application altered oat growth and rhizosphere microbial enrichment. These observations raise the possibility that IPP-associated metabolic processes are linked to plant–microbe interactions. The influence of IPP on bacterial communities was, however, strongly contingent on oat presence. The contrasting microbial responses in planted and unplanted soils indicate that the effect of exogenous IPP was strongly dependent on the presence of oat, suggesting that plant-derived processes mediated or conditioned the microbial response. In oat-planted rhizospheres, IPP apparently synergizes with root exudates to broaden ecological niche availability, fostering the coordinated growth of multiple copiotrophic phyla and significantly elevating microbial diversity (Sun et al., 2022). Together, these results position IPP as a candidate hub metabolite worthy of targeted mechanistic follow-up, rather than an established driver (**Figure 8**).

**Figure 8:**
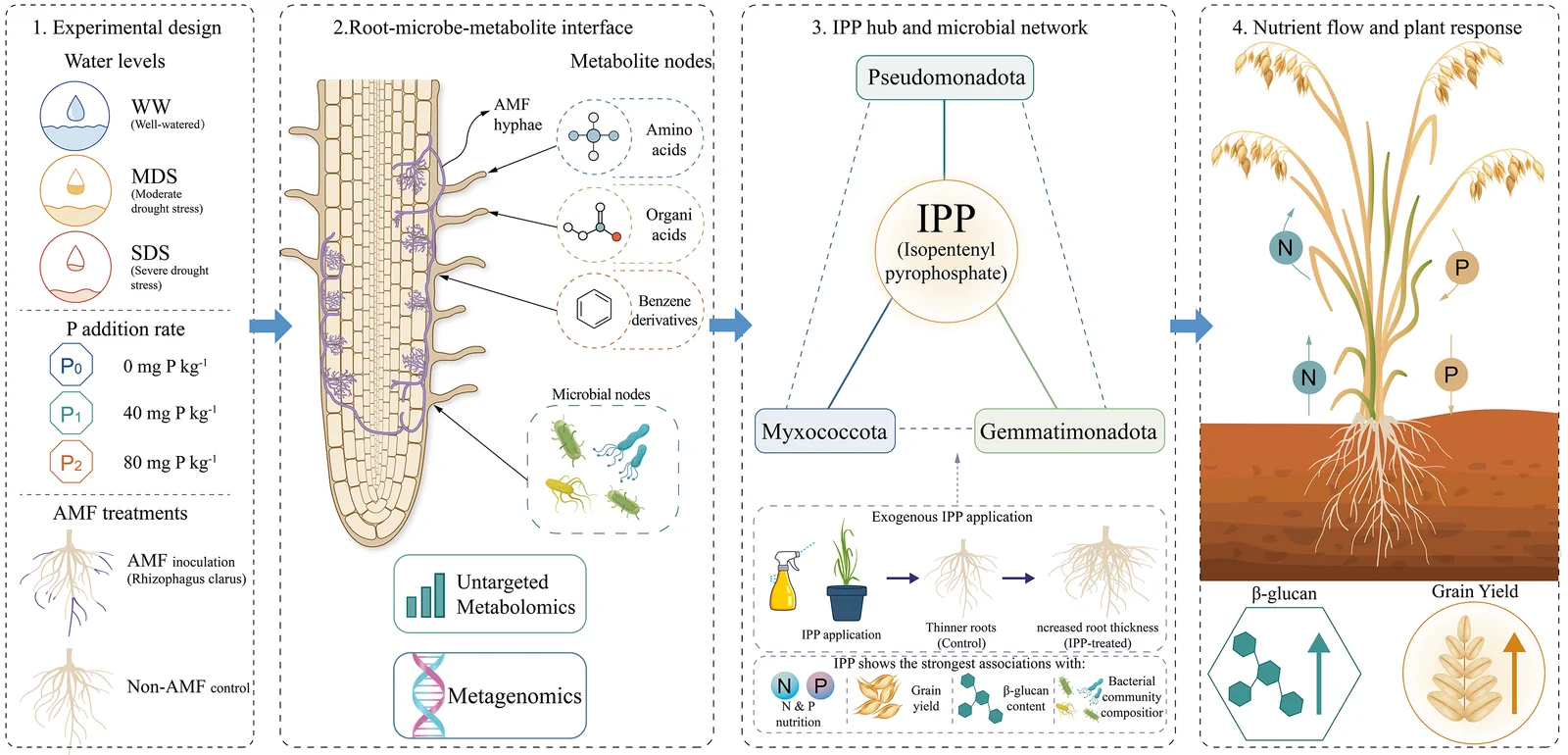
A working model summarizing the observed associations among arbuscular mycorrhizal fungi (AMF) inoculation, phosphorus (P) addition, rhizosphere metabolites, bacterial, and oat performance under drought.

Correlation analyses (**Figure 5**) revealed associations between IPP and plant–soil nutrient status, grain yield, β-glucan content, and bacterial community composition. In contrast, the exogenous IPP application experiment (**Table 1**; **Figure 6**) focused on plant growth traits and bacterial abundance under moderate drought. Accordingly, the two datasets are complementary rather than confirmatory, and the exogenous experiment should not be interpreted as direct validation of the correlative network. Rather, it was intended as an initial test of whether IPP can influence oat growth under moderate drought and to generate hypotheses for more comprehensive validation. Notably, the bacterial taxa correlated with IPP in **Figure 5** differ from those enriched by exogenous IPP in **Figure 6**. Given that root exudates are a major driver of rhizosphere microbial composition and vary across growth stages (Fan et al., 2025), and based on the results of the IPP_NP and NIPP_NP treatments without oat plants, we speculate that this mismatch may reflect the combined effects of IPP and stage-specific root exudates, rather than IPP alone. Future work will be required to examine IPP effects across the full oat growth period and under field conditions, clarify how IPP interacts with root exudates at different growth stages, and further identify and validate the core microbial taxa involved.

These results suggest that IPP may be a candidate driver of oat rhizosphere community assembly, though the specific regulatory mechanisms governing its role in oat–microbe interactions warrant dedicated follow-up investigation. It should be noted that the IPP concentration used in this study was determined through small-scale pilot screening. Although this concentration consistently promoted growth without exhibiting phytotoxicity, the absence of a concentration gradient limits the inference of thresholds, saturation points, and optimal dosages. Moreover, the lack of data on gas exchange, oxidative stress markers, nutrient uptake, and rhizosphere metabolite profiles precludes a full elucidation of the physiological mechanisms underlying IPP-promoted growth. Therefore, systematic dose–response studies, direct quantification of IPP stability and uptake kinetics (e.g., via isotope-labeled IPP tracing), and complementary measurements of the aforementioned multidimensional physiological indicators are all urgent priorities for future investigations.

## 5 Conclusion

Drought stress suppressed AMF colonization, oat grain yield, β-glucan, and WUE*_Y_* while reducing N and P concentrations across plant organs and lowering rhizosphere soil pH. AMF inoculation effectively mitigated these drought-induced impairments and, under moderate P addition, further optimized nutrient allocation during reproductive growth by elevating the total P content of spikes. Water availability was the dominant driver of both rhizosphere metabolome composition and bacterial community structure. Under drought, combined AMF inoculation and P addition enriched key metabolite classes, amino acids and derivatives, organic acids, and benzene and substituted derivatives, and was associated with shifts toward specific bacterial phyla including *Pseudomonadota*, *Myxococcota*, and *Gemmatimonadota* in the oat rhizosphere. Network analysis identified IPP as a highly connected hub metabolite within the AMF × P interaction network, showing strong statistical associations with drought-response variables, rhizosphere chemistry, bacterial community dynamics, and oat growth traits. Exogenous IPP application was associated with growth-promoting effects under controlled conditions, suggesting that IPP is a metabolite of interest for future mechanistic studies. In summary, AMF inoculation combined with appropriate P input was associated with enhanced oat yield, β-glucan accumulation, and WUE*_Y_*, alongside coordinated changes in rhizosphere metabolite and bacterial community profiles. These findings suggest a correlative framework in which IPP emerges as a candidate hub metabolite that may contribute to plant–microbe–soil interactions under drought. Future research should focus on experimentally validating the causal pathways linking IPP to rhizosphere functional microbiome assembly, and on evaluating the translational potential of IPP-related strategies for improving oat drought resilience under field conditions through integrated transcriptomic, targeted metabolomic, and functional genetic approaches.

## Data Availability

The datasets presented in this study can be found in online repositories. The names of the repository/repositories and accession number(s) can be found in the article/**Supplementary Material**.
