# Supplementary material for: Rhizophagus clarus and phosphorus co-regulate oat rhizosphere metabolome–microbiome under drought: a candidate hub metabolite perspective
Source: Front Plant Sci. 2026 Oct 21;17:1984943. doi: 10.3389/fpls.2026.1984943 (PMC13732142; doi:10.3389/fpls.2026.1984943)
Supplement: Supplementary file 1 [file DataSheet1.zip › Supplementary Material-Materials and Methods+Figures.docx]

***Supplementary Material***

1. Sample collection and measurements

*1.1. Determination and calculation of AMF infection rate*

The AMF infection rate was determined using the acetic acid ink staining method (Vierheilig et al., 1998; Zhang et al., 2025), which involves four processes: clearing, acidification, staining and decolorization. The specific methods were as follows: (1) Tender roots with a diameter of approximately 0.5 mm were placed in a small beaker and cleared in a 60 °C water bath for 20 min after the addition of 20% KOH. (2) The roots were rinsed with running water to remove KOH and then acidified with 5% acetic acid for 5 min. (3) The excess acetic acid was rinsed off, and the roots were immersed in 5% acetic acid ink staining solution without roots and placed in a 60 °C water bath for 15 min. The staining solution was then removed. (4) The roots were soaked in water and kept in a cool place for 2-3 days, during which frequent changes in soaking water occurred. The roots from each treatment were cut into approximately 1 cm segments, and 30 roots were randomly selected and placed on three slides for observation under an upright BX53 microscope (Evident, Tokyo, Japan).

*1.2.* *Rhizosphere soil and plant sample collection and measurements*

The top soil in the pot were carefully removed. Next, gently shake off the clumps of soil from the roots of each quadrat and brush down the rhizosphere soil by a sterile brush attached to the surface of the roots. Six replicate pots were sampled per treatment. After sieving through a 2-mm sieve, the 6 rhizosphere soil samples obtained from each pot as described above were mixed into one composite sample for nutrient analysis, non-targeted metabolomics, and metagenomics.

The organic matter content was determined using the potassium dichromate–external heating method. The field water-holding capacity was measured using the cutting ring method. The soil total N, ammonium N, and nitrate N were extracted with KCI using a SmarChem 200 automatic chemical analyzer (Alliance Instruments, Paris, France. The soil total P was digested with HClO_4_-H_2_SO_4_, the concentrations were subsequently determined using the Mo-Sb colorimetric method. The soil available P was determined by Olsen method using NaHCO_3_ extraction‒Mo-Sb colorimetric method. The total P of oat organs were digested with H_2_SO_4_-H_2_O_2_ and determined using Nessler's colorimetric method and vanadium molybdenum yellow spectrophotometry, respectively (Bao, 2000). The pH values were determined using a pH meter (Hanna Instruments, Woonsocket, RI, USA) with a ratio of 1 g of soil to 2.5 mL of deionized water.

2. Untargeted metabonomics analysis

*2.1 HPLC Conditions*

All samples were for two LC/MS methods. One aliquot was analyzed using positive ion conditions and was eluted from T3 column (Waters ACQUITY Premier HSS T3 Column 1.8 µm, 2.1. mm * 100 mm) using 0.1 % formic acid in water as solvent A and 0.1 % formic acid in acetonitrile as solvent B in the following gradient: 5 to 20 % in 2 min, increased to 60 % in the following 3 mins, increased to 99 % in 1 min and held for 1.5 min, then come back to 5 % mobile phase B witnin 0.1 min, held for 2.4 min. The analytical conditions were as follows, column temperature, 40 °C; flow rate, 0.4 mL/min; injection volume, 4 μL; Another aliquot was using negative ion conditions and was the same as the elution gradient of positive mode.

*2.2. MS Conditions (AB)*

The data acquisition was operated using the information-dependent acquisition (IDA) mode using Analyst TF 1.7.1 Software (Sciex, Concord, ON, Canada). The source parameters were set as follows: ion source gas 1 (GAS1),50 psi; ion source gas 2 (GAS2), 50 psi; curtain gas (CUR), 25 psi; temperature (TEM), 550 °C; decluttering potential (DP), 60 V, or−60 V in positive or negative modes, respectively; and ion spray voltage floating (ISVF), 5000 V or−4000 V in positive or negative modes, respectively. The TOF MS scan parameters were set as follows: mass range, 50–1000 Da; accumulation time, 200 ms; and dynamic background subtract, on. The product ion scan parameters were set as follows: mass range, 25–1000 Da; accumulation time, 40 ms; collision energy, 30 or−30 V in positive or negative modes, respectively; collision energy spread, 15; resolution, UNIT; charge state, 1 to 1; intensity, 100 cps; exclude isotopes within 4 Da; mass tolerance, 50 ppm; maximum number of candidate ions to monitor per cycle, 18.

3. Sequencing and analysis of 16S rRNA gene in rhizosphere soil microorganisms

Total DNA was extracted from the samples using the CretMag™ PowerSoil DNA Kit (Cretaceous, China). The quality of extracted DNA was assessed via 1% agarose gel electrophoresis, and its concentration and purity were measured using a NanoDrop 2000 spectrophotometer. The hypervariable V4 region of the bacterial 16S rRNA gene was amplified with primers 515F and 806R. PCR products from the same sample were pooled and examined by 2% agarose gel electrophoresis. Qualified amplicons were purified using the AxyPrep DNA Gel Extraction Kit (Axygen Biosciences, Union City, CA, USA). The purified products were again checked on a 2% agarose gel and quantified with a Quantus™ Fluorometer (Promega, USA). Subsequently, library construction was performed with the VAHTS® Universal Plus DNA Library Prep Kit for MGI V2 (Vazyme, China). After passing quality validation, the libraries were sequenced on the MGI sequencing platform. Raw sequencing data (raw reads) were filtered for quality using fastp (<https://github.com/OpenGene/fastp>). The filtering steps included: automatic identification and removal of adapter sequences; elimination of reads containing one or more N bases; removal of reads with more than 40% of low-quality bases (quality score < 15); deletion of sequences with an average quality below 20 within a 4-base sliding window; removal of polyG tails; and discarding of reads shorter than 150 bp. After these steps, high-quality paired-end reads (clean reads) were obtained. The high-quality paired-end reads were then assembled using FLASH (<http://ccb.jhu.edu/software/FLASH/>) to generate Clean Tags. Further, the Tag sequences were aligned against a species annotation database using vsearch (<https://github.com/torognes/vsearch/>) to detect and remove chimeric sequences, ultimately yielding the final effective data.

**Supplementary FIGURE S1** Schematic diagram of aboveground organ decomposition in oat at heading and maturity stages.

**Supplementary FIGURE S2** PCA of mass spectrometry data of each group of samples and quality control samples. The X: the first principal component, the Y: represents the second principal component, QC represents the quality control sample. WW, well-watered; MDS, moderate drought stress; SDS, serious drought stress; P0, 0 mg kg^−1^ soil; P1, 40 mg kg^−1^ soil; P2, 80 mg kg^−1^ soil; NAMF, AMF-free; AMF, AMF inoculation.

**Supplementary FIGURE S3** Category composition of metabolites in rhizosphere soil of oat.

**Supplementary FIGURE S4** PCA of oat rhizosphere soil metabolites in different treatment groups: (a) water treatment group; (b) P treatment group; (c) AMF inoculation group. The X: the first principal component, the Y: represents the second principal component. The ellipse reflects the 95 % confidence interval of samples in each group. WW, well-watered; MDS, moderate drought stress; SDS, serious drought stress; P0, 0 mg kg^−1^ soil; P1, 40 mg kg^−1^ soil; P2, 80 mg kg^−1^ soil; NAMF, AMF-free; AMF, AMF inoculation.

**Supplementary FIGURE S5** OPLS-DA score plot and validation model of rhizosphere soil metabolites of oat caused by AMF inoculation in combination with water treatment and without oat planting. Component 1 and 2 account for inter-group and intra-group variations, respectively, with their corresponding eigenvalues indicating the percentage of total variance explained in the dataset. The ellipse reflects the 95 % confidence interval of samples in each group. WW, well-watered; MDS, moderate drought stress; SDS, serious drought stress; NAMF, AMF-free; AMF, AMF inoculation; NR, without oat planting.

**Supplementary FIGURE S6** Venn diagrams (a, b) and core material classification (c) plots in different comparison groups. WW, well-watered; MDS, moderate drought stress; SDS, serious drought stress; P0, 0 mg kg^−1^ soil; P1, 40 mg kg^−1^ soil; P2, 80 mg kg^−1^ soil.

**Supplementary FIGURE S7** OPLS-DA score plot and validation model of rhizosphere soil metabolites in different treatment groups: water treatment group (a, b); phosphorus (P) treatment group (c, d); arbuscular mycorrhizal fungi (AMF) inoculation group (e, f). Component 1 and 2 account for inter-group and intra-group variations, respectively, with their corresponding eigenvalues indicating the percentage of total variance explained in the dataset. The ellipse reflects the 95 % confidence interval of samples in each group. WW, well-watered; MDS, moderate drought stress; SDS, serious drought stress; P0, 0 mg kg^−1^ soil; P1, 40 mg kg^−1^ soil; P2, 80 mg kg^−1^ soil; NAMF, AMF-free; AMF, AMF inoculation.

**Supplementary FIGURE S8** Cluster heatmap of the top 20 differential metabolites in oat rhizosphere soil in different treatment groups: water treatment group (a); phosphorus (P) treatment group (b); arbuscular mycorrhizal fungi (AMF) inoculation group (c). The heatmap displays a matrix of the relative abundance of the sample name (columns) and metabolite identifiers (rows). Group assignments are indicated by color bars, while the color gradient represents z-score normalized abundance values (red: high abundance; blue: low abundance). WW, well-watered; MDS, moderate drought stress; SDS, serious drought stress; P0, 0 mg kg^−1^ soil; P1, 40 mg kg^−1^ soil; P2, 80 mg kg^−1^ soil; NAMF, AMF-free; AMF, AMF inoculation.

**Supplementary FIGURE S9** Box plot of Chao1 (a) and Shannon (b) index based on the abundance of species in different treatment groups. WW, well-watered; MDS, moderate drought stress; SDS, serious drought stress; P0, 0 mg kg^−1^ soil; P1, 40 mg kg^−1^ soil; P2, 80 mg kg^−1^ soil; NAMF, AMF-free; AMF, AMF inoculation.

**Supplementary FIGURE S10** Correlation heatmap of gene abundance for each sample. The larger the figure of the lower triangular region, the greater the correlation, and vice versa. WW, well-watered; MDS, moderate drought stress; SDS, serious drought stress; P0, 0 mg kg^−1^ soil; P1, 40 mg kg^−1^ soil; P2, 80 mg kg^−1^ soil; NAMF, AMF-free; AMF, AMF inoculation.

**Supplementary FIGURE S11** Principal component analysis (PCA) of bacterial community at the phylum level in different treatment groups: water treatment group (a); phosphorus (P) treatment group (b); arbuscular mycorrhizal fungi (AMF) inoculation group (c). The ellipse reflects the 95% confidence interval of samples in each group. WW, well-watered; MDS, moderate drought stress; SDS, serious drought stress; P0, 0 mg kg^−1^ soil; P1, 40 mg kg^−1^ soil; P2, 80 mg kg^−1^ soil; NAMF, AMF-free; AMF, AMF inoculation.

**Supplementary FIGURE S12** The difference of the top 10 bacterial community composition at the phylum level (a, b, c) and species level (d, e, f) under water, phosphorus (P), and arbuscular mycorrhizal fungi (AMF) treatment. WW, well-watered; MDS, moderate drought stress; SDS, serious drought stress; P0, 0 mg kg^−1^ soil; P1, 40 mg kg^−1^ soil; P2, 80 mg kg^−1^ soil; NAMF, AMF-free; AMF, AMF inoculation.

**Supplementary FIGURE S13** The difference of the top 10 bacterial community composition at the phylum level (a) and species level (b) caused by AMF inoculation in combination with water treatment and without oat planting. WW, well-watered; MDS, moderate drought stress; SDS, serious drought stress; NAMF, AMF-free; AMF, AMF inoculation; NR, without oat planting.

**Supplementary FIGURE S14** LEfSe analysis of bacterial community composition in response to different treatment groups: water treatment group (a); phosphorus (P) treatment group (b); arbuscular mycorrhizal fungi (AMF) inoculation group (c). WW, well-watered; MDS, moderate drought stress; SDS, serious drought stress; P0, 0 mg kg^−1^ soil; P1, 40 mg kg^−1^ soil; P2, 80 mg kg^−1^ soil; NAMF, AMF-free; AMF, AMF inoculation.

**Supplementary FIGURE S15** Spearman correlation heatmap with hierarchical clustering showing associations between differential metabolites (columns) and differential bacterial community (row) in different comparison groups: (a), WW vs MDS vs SDS; (b), P0 vs P1; (c), P0 vs P2 (d), NAMF vs AMF. Dendrograms depict hierarchical clustering of bacterial community (left) and metabolites (top). Positive and negative correlations are indicated in red and blue, respectively. * indicates significant at the 0.05 level and ** indicates significant at the 0.01 level. WW, well-watered; MDS, moderate drought stress; SDS, serious drought stress; P0, 0 mg kg^−1^ soil; P1, 40 mg kg^−1^ soil; P2, 80 mg kg^−1^ soil; NAMF, AMF-free; AMF, AMF inoculation.

**References**

Bao, S.D. (2000). Soil agrochemical analysis. 3 edition. China agriculture press, Beijing.

Vierheilig, H., Coughlan, A.P., Wyss, U., Piché, Y. (1998). Ink and Vinegar, a Simple Staining Technique for Arbuscular-Mycorrhizal Fungi. *Appl. Environ. Microbiol.* 64, 5004-5007.

Zhang, B., Li, X., Bao, J., Tian, Z., Zhang, F., Zhang, M. (2025). A combined strategy using funneliformis mosseae and phosphorus addition for enhancing oat drought tolerance. *Agronomy* 15, 2033.
